# Percutaneous Vaccination as an Effective Method of Delivery of MVA and MVA-Vectored Vaccines

**DOI:** 10.1371/journal.pone.0149364

**Published:** 2016-02-19

**Authors:** Clement A. Meseda, Vajini Atukorale, Jordan Kuhn, Falko Schmeisser, Jerry P. Weir

**Affiliations:** Division of Viral Products, Center for Biologics Evaluation and Research, US Food & Drug Administration, 10903 New Hampshire Avenue, Silver Spring, Maryland, 20993, United States of America; Centers for Disease Control and Prevention, UNITED STATES

## Abstract

The robustness of immune responses to an antigen could be dictated by the route of vaccine inoculation. Traditional smallpox vaccines, essentially vaccinia virus strains, that were used in the eradication of smallpox were administered by percutaneous inoculation (skin scarification). The modified vaccinia virus Ankara is licensed as a smallpox vaccine in Europe and Canada and currently undergoing clinical development in the United States. MVA is also being investigated as a vector for the delivery of heterologous genes for prophylactic or therapeutic immunization. Since MVA is replication-deficient, MVA and MVA-vectored vaccines are often inoculated through the intramuscular, intradermal or subcutaneous routes. Vaccine inoculation via the intramuscular, intradermal or subcutaneous routes requires the use of injection needles, and an estimated 10 to 20% of the population of the United States has needle phobia. Following an observation in our laboratory that a replication-deficient recombinant vaccinia virus derived from the New York City Board of Health strain elicited protective immune responses in a mouse model upon inoculation by tail scarification, we investigated whether MVA and MVA recombinants can elicit protective responses following percutaneous administration in mouse models. Our data suggest that MVA administered by percutaneous inoculation, elicited vaccinia-specific antibody responses, and protected mice from lethal vaccinia virus challenge, at levels comparable to or better than subcutaneous or intramuscular inoculation. High titers of specific neutralizing antibodies were elicited in mice inoculated with a recombinant MVA expressing the herpes simplex type 2 glycoprotein D after scarification. Similarly, a recombinant MVA expressing the hemagglutinin of attenuated influenza virus rgA/Viet Nam/1203/2004 (H5N1) elicited protective immune responses when administered at low doses by scarification. Taken together, our data suggest that MVA and MVA-vectored vaccines inoculated by scarification can elicit protective immune responses that are comparable to subcutaneous vaccination, and may allow for antigen sparing when vaccine supply is limited.

## Introduction

The eradication of smallpox, a disease that caused the death of hundreds of millions of people over many centuries, was accomplished primarily by the use of replication-competent vaccinia virus strains as vaccines. Traditional (first-generation) smallpox vaccines, as well as more recently developed cell culture-derived second-generation smallpox vaccines such as ACAM2000 [1,2], the currently licensed smallpox vaccine in the United States, are inoculated into vaccine recipients by scarification of the skin surface, also known as percutaneous, skin or cutaneous vaccination [3]. Rare but severe adverse reactions caused by these vaccines, including generalized vaccinia, eczema vaccinatum, and the more recently recognized cases of myopericarditis [4,5,6,7], limit the use of these vaccines for routine preventative vaccination of the general populace in the absence of any immediate risk of exposure to variola virus (the etiologic agent for smallpox) or other pathogenic orthopoxviruses such as monkeypox virus.

Thus, as early as the 1930s, efforts were made to develop safer smallpox vaccines by attenuating existing strains of vaccinia virus [8,9]. As part of this effort, the modified vaccinia virus Ankara (MVA) was developed in the early 1970s. MVA was derived from the chorioallantois vaccinia virus Ankara (CVA) strain of vaccinia virus, by more than 570 passages in chick embryo fibroblast (CEF) cells [10]. During the course of passage of CVA in CEF cells, several genes (mainly host-range and immunomodulatory genes) were lost, resulting in the severely attenuated MVA. About 15% of the viral genome was lost during passage in CEF cells, and MVA does not replicate productively in most mammalian cells [11,12,13]. MVA has been extensively evaluated in different animal models [14,15,16,17] and in clinical trials, and found to be less reactogenic when compared with replication-competent first and second generation smallpox vaccines [18,19]. MVA is licensed as a smallpox vaccine in Europe and Canada, and currently undergoing clinical development in the United States.

The severe attenuation of MVA and its consequent loss of the capacity to replicate efficiently in mammalian cells is evident in its inability to produce a “vaccine take”, a pustular lesion that develops at the inoculation site, when vaccinia virus is inoculated on the skin surface. Apart from its potential use as a smallpox vaccine in immunocompromised individuals, MVA has the capacity to accommodate heterologous DNA, and express encoded proteins, thus serving as a useful viral vector in vaccine development against different types of pathogens. Several recombinant MVA vectors expressing heterologous proteins of different human pathogens are at various phases of clinical development [20,21] Some of the MVA-vectored vaccines in clinical trials include those expressing human immunodeficiency virus antigens [22,23, 24], Mycobacterium tuberculosis 85A antigen [25,26,27], malaria antigens [28,29,30], human papilloma virus antigen [31], hepatitis C antigens [32,33], respiratory syncytial virus antigens [34], influenza virus antigens [35,36,37], Epstein-Barr virus antigen [38,39] and more recently, ebola virus antigens [40]. Several other MVA-vectored vaccines have also been evaluated in preclinical studies [41,42,43]. In most preclinical and clinical studies, MVA or recombinant MVA vectors, unlike replication-competent vaccinia virus strains, are inoculated into subjects via the intramuscular, intradermal or subcutaneous routes. Although MVA has been demonstrated to have a better safety profile than replication-competent vaccinia virus, a relatively large inoculum volume of 0.05 to 0.10mL and 0.5 to 1mL of MVA or recombinant MVA is typically used in small animal models and in non-human primates/humans, respectively. This often results in local reactions at the site of inoculation, including muscle ache, pain, and tenderness [44,36]. In addition, the inoculation of prophylactic or therapeutic regimens with needles and syringes can be problematic for some people, a global problem commonly called needle phobia or fear of the needle in common phraseology. Although the use of needle-free injection devices such as the jet injector [45] has become increasingly popular, hypodermic syringes and needles remain in wide use for the delivery of prophylactic and therapeutic remedies.

Previously, we [46] described a recombinant vaccinia virus, A33R/B5Rko, that was severely attenuated for plaque formation in permissive cell lines due to deletions of the *A33R* and *B5R* genes, encoding the A33 and B5 proteins, respectively, of the extracellular virion form of vaccinia virus. The severe attenuation and growth characteristics of the A33/B5Rko virus is reminiscent of the properties of MVA. A33R/B5Rko elicited vaccinia-specific IgG and neutralizing antibodies when BALB/c mice were inoculated by scarification at the base of the tail. In an intranasal challenge model, using the Western Reserve strain of vaccinia virus, the A33R/B5Rko virus conferred comparable levels of protection on mice as those vaccinated with a clonal isolate of Dryvax, DV-3.

During the course of the work above [46], antibody [47] and robust cell-mediated immune responses after the inoculation of recombinant MVA vectors through the skin, were also reported [48,49,50], suggesting that percutaneous inoculation of MVA may elicit equivalent or higher immune responses than subcutaneous injection. In the work described here, we demonstrate in mouse models that percutaneous inoculation of MVA elicited protective immune responses against lethal intranasal challenge with the Western Reserve (WR) strain of vaccinia virus, and at low doses of MVA, lower morbidity was recorded in mice that were vaccinated via the percutaneous route than in those immunized via the intramuscular or subcutaneous routes. In addition, we show in two models that percutaneous inoculation of recombinant MVA expressing heterologous antigens elicited specific immune responses, including neutralizing antibodies, at levels that are comparable to subcutaneous inoculation. A recombinant MVA expressing herpes simplex virus 2 (HSV-2) glycoprotein D elicited gD2-specific IgG and HSV-2 neutralizing antibodies, and a recombinant MVA expressing the hemagglutinin of influenza rgA/Vietnam/1203/04, H5N1, elicited protective immune responses when inoculated by the percutaneous route. Our data suggest that vaccination via the percutaneous route is efficient in stimulating protective immune responses, and may find clinical relevance in immunizations with MVA and MVA-vectored vaccines.

## Materials and Methods

### Viruses

The modified vaccinia virus Ankara (MVA; ATCC # VR-1508) was provided by Drs. Linda Wyatt and Bernard Moss (NIAID/NIH) and vaccinia virus strain Western Reserve (VV-WR; ATCC # VR-1354) was provided by Dr. Bernard Moss. We have previously described the construction of a recombinant MVA expressing the herpes simplex virus type 2 glycoprotein D (MVA-gD2), in which the Us6 gene of HSV-2 (strain MS) was inserted into the deletion II region in MVA by homologous recombination [51]. Expression of glycoprotein D is driven by the synthetic vaccinia early/late promoter [52]. Recombinant MVA expressing the hemagglutinin (H5) of influenza virus rgA/Viet Nam/1203/2004 (H5N1) (MVA-HA) was constructed by homologous recombination of the H5 gene into the deletion III region of MVA [53]. Expression of the influenza H5 is driven by the vaccinia *H5R* promoter. MVA and recombinant MVA vectors were prepared from primary chick embryo fibroblast cells (CEF) or DF-1 cells (ATCC # CRL-12203) that had been infected with the appropriate virus as previously described [54], and partially purified viruses were obtained by passing infected cell lysates through a 36% sucrose cushion. MVA and MVA recombinants were titered on DF-1 cell monolayers. VV-WR was prepared from infected BSC-1 cells (ATCC # CCL-26), and was also partially purified through a 36% sucrose cushion, and titered on BSC-40 cell monolayers (ATCC # CRL-2761). The attenuated influenza virus rgA/Viet Nam/1203/2004 (H5N1) was grown in the allantoic cavity of embryonated eggs and titrated on MDCK cells. Cells were cultivated and regularly maintained in DMEM containing 10% fetal bovine serum (5% for MDCK cells) and 5μg/mL gentamicin.

### Mice

BALB/c mice (4–5 weeks old) were obtained from the Jackson Laboratories, Bar Harbor, Maine. Mice received feed and water freely. The animal study protocol for the work described in this manuscript was approved by the Institutional Animal Care and Use Committee (IACUC) of the Center for Biologics Evaluation and Research, USFDA (CBER/FDA).

### Animal inoculation and virus challenge

Intramuscular inoculation into the muscles of the hind legs and subcutaneous inoculation at the base of the tail using 1mL tuberculin syringes affixed with 25-gauge needles, were performed as previously described [17]. Tail scarification of anesthetized animals was also performed as previously described [17]. Briefly, the inoculum was diluted in sterile endotoxin-free phosphate-buffered saline (PBS) such that the desired unit dose is contained per 2μL. Mice were anesthetized by intraperitoneal injection of 1x avertin (2,2,2,-Tribromoethanol in *tert*-Amyl alcohol (Sigma-Aldrich, St. Louis, Missouri)) diluted in PBS at 20 μL per gram body weight. Using a 25-gauge needle, 15 to 20 needle scratches/puncture were made at the base of the tail and 2μL of the inoculum was applied to the scarified surface.

Vaccinia virus (strain WR) and attenuated influenza A virus (rgA/Viet Nam/1203/2004 (H5N1)), a 6+2 reassortant vaccine strain bases on influenza A/Puerto Rico/8/34, were used in challenge experiments. For intranasal mouse challenge, a dose of ten 50% lethal dose (10 LD_50_) (equivalent to 1.5 x 10^5^ pfu) of VV-WR was used. For challenge with rgA/Viet Nam/1203/2004 (H5N1)), a dose of 10^6^ pfu was used. We previously determined this dose to be lethal in our influenza virus challenge model. Mice were anesthetized by intraperitoneal injection of 1x avertin (20 μL per gram body weight), followed by inoculation of the challenge virus in a total of 20 μL suspension into the nares (10μL per naris) as previously described [17]. Mice were monitored and weighed daily for two weeks post challenge. Mice that lost 25% of their initial body weight were considered to have reached the study endpoint, and were humanely euthanized per the IACUC-approved animal study protocol.

### IgG ELISAs and assays for neutralizing antibodies

Testing of antisera for antigen-specific antibodies (IgG) was performed by standard enzyme-linked immunosorbent assay (ELISA). ELISA for vaccinia-specific antibodies was performed using psoralen/UV-inactivated vaccinia virus (Dryvax) as the antigen, as previously described [17]. HSV-2 gD-specific IgG ELISA using partially-purified HSV-2 glycoproteins (50 ng/well) as the antigen, and HSV-2 neutralization assay were performed in 96-well Immulon 2^HB^ plates (Fisher Scientific) and 96-well tissue culture plates (Corning), respectively, as previously described [51]. Influenza virus H5-specific IgG was quantified using Sanofi Pasteur’s inactivated rgA/Viet Nam/1203/2004 vaccine (CBER reference antigen #50) as the coating antigen. Immulon 2^HB^ plates were coated with CBER reference antigen at 1 μg/well, and plates were stored at 4°C overnight. Plates were washed with phosphate-buffered saline containing 0.5% Tween-20 (PBST), and then blocked with PBST containing 10% fetal bovine serum for 2 hours. Subsequent incubation of the antigen with the test serum samples and completion of the assay followed standard ELISA protocol as previously described [51].

Testing of antisera for the neutralization of influenza A virus rgA/Viet Nam/1203/2004 (H5N1), an attenuated vaccine strain generated by reverse genetics, was performed using a microneutralization assay as previously described [55]. The presence of virus was detected using biotin-conjugated antibody to influenza NP (clone A; Milipore, Billerica, MA, USA) followed by HRP-labeled Streptavidin (KPL, Gaithersburg, MD, USA).

### Cytokine assay

Mice were vaccinated with 10^7^ pfu of MVA-gD2 via the subcutaneous or percutaneous route. A control group was vaccinated with 10^7^ pfu of MVA. Seven (7) days after vaccination, mice were euthanized, spleens were collected from dissected mice, and lymphocytes were isolated from spleen homogenates as previously described [51]. Spleen cells were cultured in 24-well tissue culture plates and re-stimulated with live HSV-2 (strain MS) at a multiplicity of infection of 1.0. Culture supernatants were harvested after 48 hours, clarified of cells by centrifugation, and tested for IL-2 and IFN-γ by capture ELISA as described [51], and reagents obtained from BD Pharmingen.

### Statistical analysis

Unpaired, two-tailed Student’s *t-test* for statistical comparison of antibody titers was performed using GraphPad Prism 6.02 software (GraphPad Software, Inc.). The Kaplan-Meier Survival LogRank test was performed using the SigmaPlot 13.0 software (Systat Software, Inc.), and was used to compare differences in the number of surviving animals in the various treatment groups after virus challenge. In all cases, a *p* value <0.05 indicates statistically significant differences between treatment groups.

## Results

### Percutaneous inoculation of MVA elicits vaccinia-specific antibody responses and protects against VV-WR challenge

In a preliminary experiment to investigate the utility of the percutaneous route for the delivery of MVA, we observed that MVA delivered by tail scarification, while statistically insignificant (*p* = 0.298), elicited a higher vaccinia-specific IgG response and protection in mice than the same dose (10^6^ pfu) delivered by the intramuscular route (S1 Fig). The experiment using 10^6^ pfu of MVA did not allow us to observe any differences in antibody response, disease progression and protection between the two immunization routes. Thus in subsequent experiments, lower doses of MVA were evaluated.

**Fig 1:**
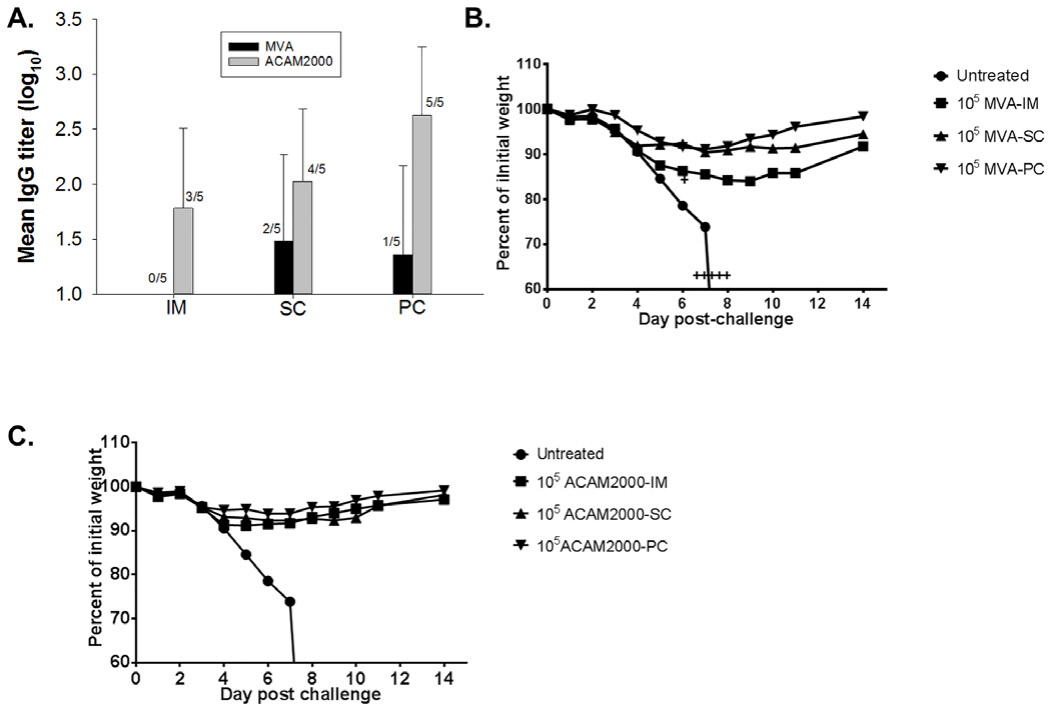
Immunogenicity and protection conferred by MVA and ACAM2000. Groups of mice were vaccinated with 10^5^ pfu of MVA or ACAM2000. Each virus was administered at the indicated dose via the intramuscular (IM), subcutaneous (SC), or percutaneous (PC) routes. Antisera were obtained from mice after three weeks and tested for vaccinia-specific IgG by ELISA. The mean IgG titers are presented (**A**). For computational purposes, a mouse with below detection level of IgG was assigned a titer of 1.0 (log_10_). Fractions indicate the number of seropositive animals in each group, with error bars representing standard deviation. Mice were challenged intranasally with 10 LD_50_ (1.5 x 10^5^ pfu) of vaccinia virus (strain Western Reserve). Morbidity, as measured by mean weight changes post-challenge, is shown for the MVA treatment groups (**B**), and for the ACAM2000 treatment groups (**C**). The “Untreated” control group was the same for both the MVA and ACAM2000 groups. A “**+**” sign represents a mouse that succumbed to infection.

Groups of mice were vaccinated with 10^5^ pfu of MVA via the intramuscular, subcutaneous, or percutaneous routes. For comparison, a set of three groups of mice were similarly vaccinated with 10^5^ pfu of the licensed ACAM2000 smallpox vaccine, via the same three routes. An untreated group was included as a control. Three weeks after vaccination, serum samples were obtained from all mice and tested for vaccinia-specific antibodies by ELISA, using inactivated Dryvax as the antigen. Among mice in the MVA treatment cohort, an IgG response was detectable in 2/5 mice in the subcutaneous group, and 1/5 in the percutaneous group. The untreated mice and all mice in the MVA intramuscular group had no detectable IgG at this time point. By contrast, all but three mice (2/5 and 1/5 in the intramuscular and subcutaneous groups, respectively) in the ACAM2000 cohort had detectable levels of IgG (Fig 1A). At 4 weeks post-immunization, all mice were challenged intranasally, with a lethal dose (10 LD_50_) of VV-WR. All mice in the untreated group showed severe symptoms of infection, reaching the study endpoint of 25% weight loss by day 7 post-challenge, and had to be euthanized (Fig 1B & 1C). Except for a mouse in the MVA-intramuscular group, all other mice vaccinated with MVA or ACAM2000, survived. Among the MVA vaccinated animals, mice in the intramuscular group lost the most weight (Fig 1B), with a mean peak loss of about 16% on day 9 post-challenge. Weight loss among the MVA subcutaneous and percutaneous groups were similar, with peak losses of 9.6% (day 6) and 8.9% (day 7), respectively. However, mice in the MVA percutaneous group recovered more quickly (98.4% of original mean body weight on day 14) than the subcutaneous group (94.4% mean weight on day-14). Among the ACAM2000 treatment groups (Fig 1C), the average peak weight loss was 8.8% (day-5), 7.7% (day-6), and 6.2% (day-6), for the intramuscular, subcutaneous, and percutaneous groups, respectively. In another experiment, mice in groups of five were vaccinated with 10^3^ pfu or 10^5^ pfu of MVA by scarification. Two other groups were similarly vaccinated with 10^3^ pfu or 10^5^ pfu of ACAM2000, and a control group was scarified with PBS. Antisera were collected after three weeks and mice were challenged with 10 LD_50_ of VV-WR. None of the mice in the PBS group had detectable IgG and all succumbed to VV-WR infection (Table 1). All mice in the 10^3^ pfu MVA group had no detectable IgG and 2/5 in the 10^5^ MVA group had detectable IgG. However, 1/5 and 5/5 of mice survived in the 10^3^ pfu and 10^5^ pfu MVA, respectively. In the ACAM2000 cohort, 2/5 and 5/5 of mice in the 10^3^ pfu and 10^5^ pfu groups, respectively, were seropositive. Three of five (3/5) and 5/5 of mice in the ACAM2000 cohort survived VV-WR challenge (Table 1). These sets of data suggest that in this mouse model, MVA inoculation by the percutaneous route elicits equivalent or greater protective immune responses than inoculation via the intramuscular or subcutaneous routes.

**Table 1:** Antibody response and protection after MVA and ACAM2000 inoculation by tail scarification.

Group	PBS	10^3^ pfu MVA	10^5^ pfu MVA	10^3^ pfu ACAM	10^5^ pfu ACAM
Seropositivity	0/5	0/5	2/5	2/5	5/5
Log_10_ mean IgG titer (± SD)	<LOQ	<LOQ	1.36 (± 0.49)	1.48 (± 0.66)	2.08 (± 0.16)
Survival	0/5	1/5	5/5	3/5	5/5

LOQ = limit of quantitation (0.3 log_10_); ACAM = ACAM2000 smallpox vaccine. In calculating mean IgG, a log_10_ titer of 1.0 was assigned to serum samples with no detectable IgG.

### Percutaneous vaccination with recombinant MVA-gD2 elicits HSV-2-specific immune responses

**Fig 2:**
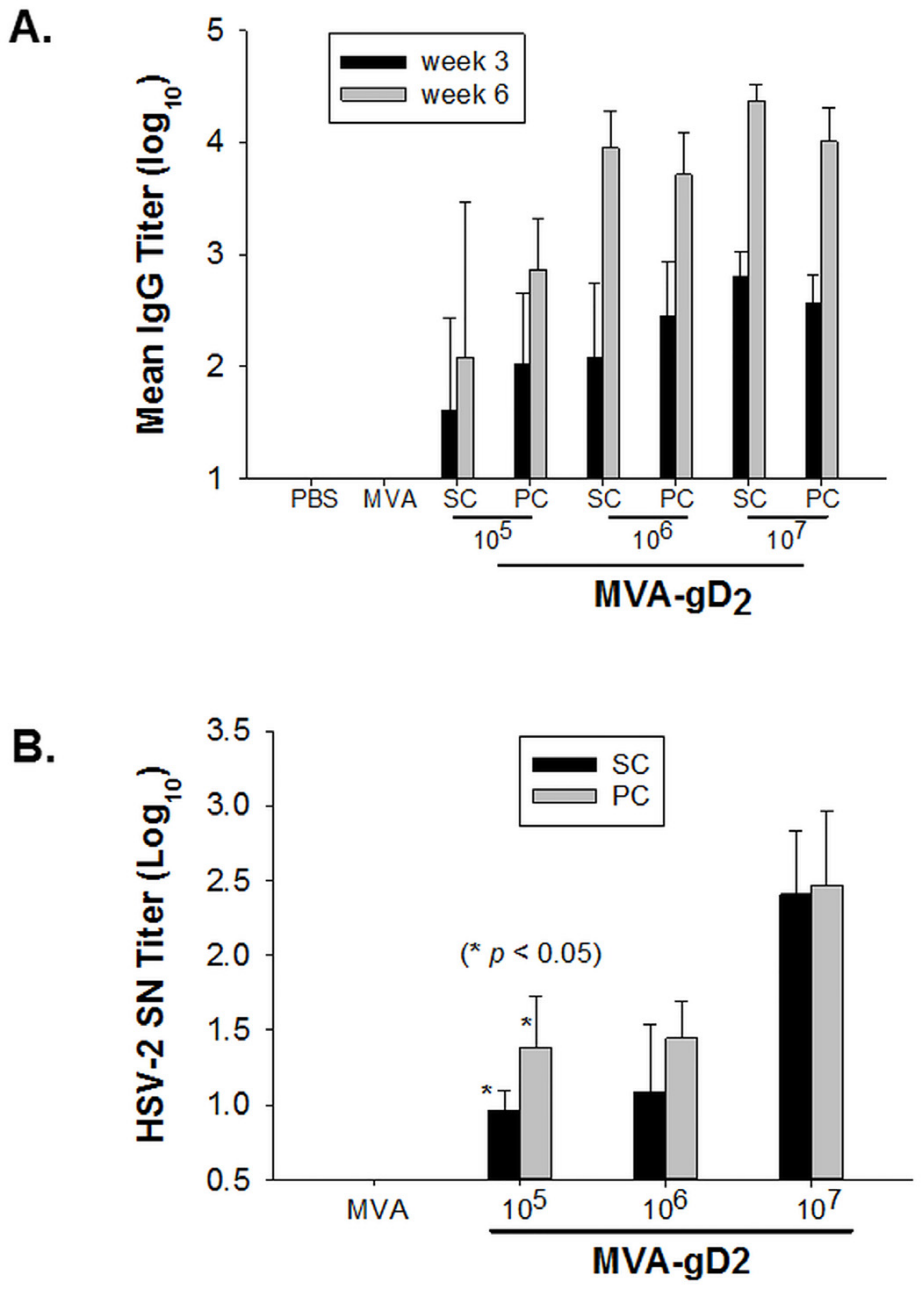
Antibody response to MVA-gD2 inoculated by the subcutaneous or percutaneous route. Groups of mice were vaccinated with MVA-gD2 at doses of 10^5^, 10^6^, or 10^7^ pfu, on a prime-boost schedule, with an interval of three weeks between the two vaccinations. A control group was vaccinated with 10^7^ pfu of MVA subcutaneously. Antisera obtained from mice 3 weeks after the first vaccination (week 3) and 3 weeks after the second vaccination (week 6) were tested for HSV-2 gD-specific IgG by ELISA. The log_10_ values of the mean IgG titers are shown in **A**. The week-6 antisera were also tested for the neutralization of HSV-2 infectivity in Vero cells. Mean serum neutralization (SN) antibody titers are shown in **B**. Error bars represent standard deviation.

MVA is an attractive vector being used for the expression of transgenes and has been used in the expression of antigens of a variety of pathogens. Similar to studies investigating MVA as a smallpox vaccine, preclinical and clinical evaluation of MVA-vectored vaccines in development has relied predominantly on the use of intramuscular, intradermal or subcutaneous routes of MVA delivery. In order to determine whether the percutaneous route will be useful for the evaluation of MVA-vectored recombinant vaccines, a recombinant MVA (MVA-gD2) expressing the glycoprotein D of herpes simplex virus type-2 (HSV-2, strain MS) was evaluated for immunogenicity in the BALB/c mouse model. In this recombinant MVA, the HSV-2 Us6 gene encoding glycoprotein D was inserted into the deletion II site in MVA by homologous recombination [51]. Groups of mice were vaccinated with MVA-gD2 at three dose levels: 10^5^ pfu, 10^6^ pfu, and 10^7^ pfu; with each dose level administered subcutaneously or percutaneously. The control group received 10^7^ pfu of MVA subcutaneously. All treatment groups were vaccinated using a prime/boost immunization strategy as previously described [51]. Antisera were collected 3 weeks after the priming vaccination, as well as at 3 weeks after the boost, and were tested for HSV-2-specific IgG at both time points, using partially-purified HSV-2 glycoproteins as the antigen (Fig 2A). Antisera collected at the 6-week time point were also tested for HSV-2 neutralizing antibodies (Fig 2B). Antisera obtained from mice vaccinated with the MVA vector had no detectable HSV-2 specific antibodies at both time points. HSV-2 specific antibodies were detected in antisera obtained from mice vaccinated with MVA-gD2 at both time points (Fig 2A). The mean IgG titers (log_10_) after the priming vaccinations were 1.60 (±0.82 standard deviation (SD)), 2.08 (±0.66 SD), and 2.81 (±0.21 SD), for the 10^5^ pfu, 10^6^ pfu, and 10^7^ pfu, respectively, in the MVA-gD2 subcutaneous cohort. In the three dosage groups vaccinated via the percutaneous route, mean IgG titers (log_10_) of 2.02 (±0.62 SD), 2.44 (±0.49 SD), and 2.56 (±0.25 SD), were obtained, respectively. Two of five (2/5) mice and a mouse (1/5) in the 10^5^ pfu and 10^6^ pfu subcutaneous groups, respectively, as well as a mouse (1/5) in the 10^5^ pfu percutaneous group did not seroconvert three weeks after the priming vaccination. Higher antibody titers were obtained across the board in all MVA-gD2 groups following booster vaccinations. Thus, at the 6-week time point (i.e., 3 weeks after booster vaccination), mean IgG titers of 2.08 (±1.39 SD), 3.95 (±0.33 SD), and 4.37 (±0.13 SD) were recorded for the 10^5^ pfu, 10^6^ pfu, and 10^7^ pfu, respectively, in the MVA-gD2 subcutaneous cohort. Two of five (2/5) mice in the 10^5^ pfu subcutaneous group still failed to seroconvert after booster vaccination. All mice in the percutaneous vaccination groups seroconverted at the 6-week time point. At this time point, the mean IgG titers (log_10_) for the three percutaneous groups (10^5^ pfu, 10^6^ pfu, and 10^7^ pfu) were 2.87 (±0.45 SD), 3.71 (±0.37 SD), and 4.01 (±0.30 SD), respectively.

The serum samples obtained at the 6-week time point were tested for the ability to neutralize HSV-2 infectivity in Vero cells. Similar to the IgG ELISA result, antisera from the MVA control group did not neutralize HSV-2 (mean serum neutralization (SN) titer was below the level of quantitation of 0.30 log_10_). Low to modest neutralizing antibody titers were detected in MVA-gD2 vaccinated mice (Table 2).

**Table 2:** Neutralizing antibody titers and cytokine responses following subcutaneous and percutaneous vaccinations with MVA-gD2.

Group	10^7^ MVA	10^5^ MVA-gD2-SC	10^5^ MVA-gD2-PC	10^6^ MVA gD2-SC	10^6^ MVA-gD2-PC	10^7^ MVA-gD2-SC	10^7^ MVA-gD2-PC
**SN Titer, Log**_**10**_ **(±SD)**	<0.30	0.96 (±0.13)	1.38 (±0.34)	1.08 (±0.46)	1.44 (±0.25)	2.41 (±0.43)	2.47 (±0.49)
**IL-2 (pg/mL ±SD)**	< LOQ	NT	NT	NT	NT	40.3 (± 21)	52.3 (± 40.1)
**IFN-γ (pg/mL ±SD)**	< LOQ	NT	NT	NT	NT	28.5 (±17.4)	82.3 (± 114.3)

LOD = limit of quantitation (0.3 log_10_); SC = subcutaneous; PC = percutaneous; SD = standard deviation; N/A = not applicable.

In the subcutaneous vaccination sub-groups, mean SN titers (log_10_) of 0.96 (±0.13), 1.08 (±0.46), and 2.41 (±0.43), were obtained for the 10^5^ pfu, 10^6^ pfu, and 10^7^ pfu groups, respectively. The SN titers for the three dosages in the percutaneous groups, were 1.38 (±0.34), 1.44 (±0.25), and 2.47 (±0.49), respectively.

In another experiment, cellular immune response to MVA-gD2 was evaluated. Mice were vaccinated with 10^7^ pfu of MVA subcutaneously, or with 10^7^ pfu of MVA-gD2 either subcutaneously or percutaneously. Spleen cells were harvested from mice 7 days after vaccination and tested for cytokine (IL-2 and IFN-γ) secretion as previously described (Meseda et al., 2002). spleen cells were re-stimulated *in vitro* by live infection with HSV-2 (strain MS) at a multiplicity of infection of 1.0. Supernatants were collected from the cultured spleen cells and tested for levels of IL-2 and IFN-γ. Whereas the levels of IL-2 and IFN-γ in the supernatants from the spleen of MVA-infected mice were below detection, all mice in both MVA-gD2 groups had detectable levels of IL-2 and IFN-γ, (Table 2). Mean IL-2 levels of 40.3 ± 21 pg/mL and 52.3 ± 40.1 pg/mL were obtained for the MVA-gD2 subcutaneous and percutaneous groups, respectively. Similarly, IFN-γ levels were 28.5 ± 17.4 pg/mL, and 82.3 ± 114.3 pg/mL, for the subcutaneous and percutaneous MVA-gD2 groups, respectively. Taken together, this set of data suggests that the inoculation of recombinant MVA-gD2 by scarification is capable of eliciting antigen-specific immune responses that are comparable or higher than delivery by subcutaneous inoculation.

### Percutaneous vaccination with MVA-HA elicits protective immunity against virus challenge

MVA is used as a vector for the expression of heterologous antigens. The deletion sites in MVA, including the deletion II and deletion III sites, are commonly used for the insertion of transgenes. In order to broaden our understanding of the utility of the percutaneous route for the delivery of MVA-vectored vaccines, we further evaluated a recombinant MVA, MVA-HA, in which the hemagglutinin gene of influenza A virus rgA/Viet Nam/1203/2004 (H5N1), was inserted in the deletion III site of MVA. In a series of experiments, the antibody response following vaccination via the subcutaneous or percutaneous routes was characterized, and the protective effectiveness of vaccination via these routes was evaluated in a mouse intranasal challenge model.

In the first experiment, groups of mice (five per group) were vaccinated with 10^5^, 10^6^, or 10^7^ pfu of MVA-HA on a prime-boost schedule at an interval of 3 weeks between vaccinations. Each dose of MVA-HA was administered via the subcutaneous or percutaneous routes. A control group was vaccinated with 10^7^ pfu of MVA via the subcutaneous route. Three weeks after vaccination, serum samples were obtained from mice and tested for H5-specific IgG, and all mice were challenged with 10^6^ pfu of attenuated influenza rgA/Viet Nam/1203/2004 (H5N1) via the intranasal route. Except for a mouse in the MVA control group that had a low level of non-specific IgG, all other mice in the control group had no H5-specific IgG, and 4/5 (including the one with detectable IgG) succumbed to influenza virus challenge. All mice that were vaccinated with MVA-HA, irrespective of the route, had high levels of H5-specific IgG titers (S2A Fig), and were protected from influenza virus challenge (S2B Fig).

**Fig 3:**
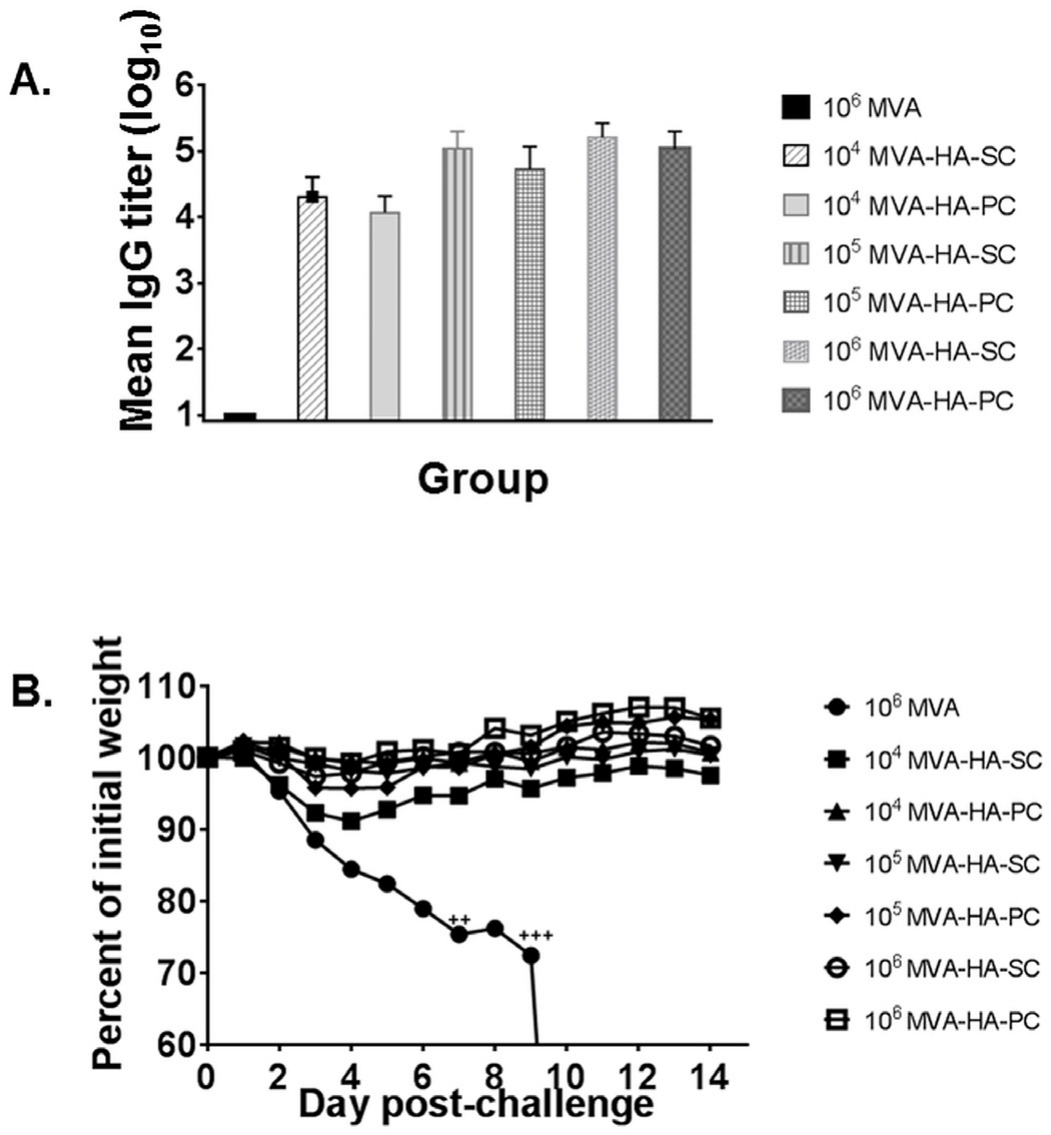
Antibody responses and protection conferred by MVA-HA inoculated via the subcutaneous or percutaneous route. Mice in groups of five were vaccinated with 10^4^, 10^5^, or 10^6^ pfu of MVA-HA via the SC and PC routes. A control group (5 mice) was vaccinated with 10^6^ pfu of MVA. Mice were vaccinated twice at an interval of three weeks between vaccinations. Post-vaccination antisera obtained 3 weeks after the boost were tested for H5-specific IgG by ELISA. The mean IgG titers are presented (**A**), with error bars representing standard deviation. Mice were challenged with 10^6^ pfu of rgA/Viet Nam/1203/2004. Weight changes (%) post-challenge are shown in (**B)**. A “**+**” sign represents a mouse that succumbed to infection.

In the second experiment, mice in groups of mice (five per group) were vaccinated with 10^4^, 10^5^, or 10^6^ pfu of MVA-HA via the subcutaneous or percutaneous route on a prime-boost schedule at an interval of 3 weeks between vaccinations. A control group was vaccinated with 10^6^ pfu of MVA, subcutaneously. Antisera were obtained from mice three weeks after the booster vaccination, and all mice were challenged with 10^6^ pfu of influenza rgA/Viet Nam/1203/2004. All mice in the MVA group had no detectable H5-specific IgG. By contrast, all mice inoculated with MVA-HA, irrespective of the dose, had high titers of H5-specific IgG (Fig 3A). The differences in IgG levels between the subcutaneous and percutaneous cohorts at each dose level of MVA-HA were not statistically significant, although mean IgG titers were slightly higher in the subcutaneous cohort. Following intranasal challenge with influenza rgA/Viet Nam/1203/2004, all mice in the MVA group succumbed to infection, and all mice vaccinated with MVA-HA, irrespective of the dose, survived with varying degrees of morbidity. Among MVA-HA vaccinated mice, the 10^4^ pfu/subcutaneous group recorded the severest weight loss (Fig 3B), and the difference in weight changes between the subcutaneous and percutaneous groups vaccinated with 10^4^ pfu was statistically significant (two-tailed p-value = <0.001).

A summary of the neutralizing antibody titers of pooled antisera for each treatment group from the two MVA-HA experiments described above is presented in Table 3. The MVA control group had no detectable neutralizing antibody in the serum samples obtained at any time point. Among the groups vaccinated with MVA-HA, neutralizing antibody was below detection in post-prime antisera, except in the 10^7^ pfu subcutaneous group where a titer of 10 was obtained. However, following the administration of booster inoculations, all MVA-HA treatment groups had detectable levels of neutralizing antibody that increased with increase in vaccine dose.

**Table 3:** Neutralizing antibody titers against the H5 of attenuated influenza rgA/Viet Nam/1203/2004, H5N1 virus.

Treatment (pfu)	Serum Neutralization Titer		
	E1-post dose 1	*E1-post dose 2	*E2-post dose 2
10^6^ MVA—SC	N/A	N/A	< 10
10^7^ MVA—SC	NT	< 10	N/A
10^4^ MVA-HA—SC	N/A	N/A	10
10^4^ MVA-HA—PC	N/A	N/A	20
10^5^ MVA-HA—SC	< 10	15	40
10^5^ MVA-HA—PC	< 10	15	20
10^6^ MVA-HA—SC	< 10	40	60
10^6^ MVA-HA—PC	< 10	60	40
10^7^ MVA-HA—SC	10	80	N/A
10^7^ MVA-HA—PC	< 10	80	N/A

NT = Not tested; N/A = not applicable; E1-post dose 1 = experiment 1,post prime antisera; E1-post dose 2 = experiment 1, post boost antisera; E2-post dose 2 = experiment 2 post boost antisera;

*titer represents the average of two repeat testing.

For each treatment group, serum samples (5 per group) were pooled for neutralization test.

Neutralizing antibody titers in mice in the subcutaneous cohort were 15, 40, and 80, for the 10^4^, 10^5^, and 10^6^ pfu, respectively. Similarly, neutralizing antibody titers among the percutaneous cohort were 15, 60, and 80, for the 10^4^, 10^5^, and 10^6^ pfu, respectively. Interestingly, the levels of neutralizing antibody for the percutaneous treatment groups were similar to the subcutaneous group at the same MVA-HA dosage, in spite of slightly higher total H5-specific IgG levels in the subcutaneous cohort (difference is not statistically significant). This set of data suggests that the antibody response, both total IgG and neutralizing antibodies, elicited by MVA-HA was comparable between mice that were inoculated via the subcutaneous route and those inoculated via the percutaneous route. Further, we observed that all mice inoculated with ≥10^4^ pfu of MVA-HA by prime-boost, were protected from influenza virus challenge.

### Discrimination of the protective effectiveness of MVA-HA inoculated via the subcutaneous or percutaneous routes

**Fig 4:**
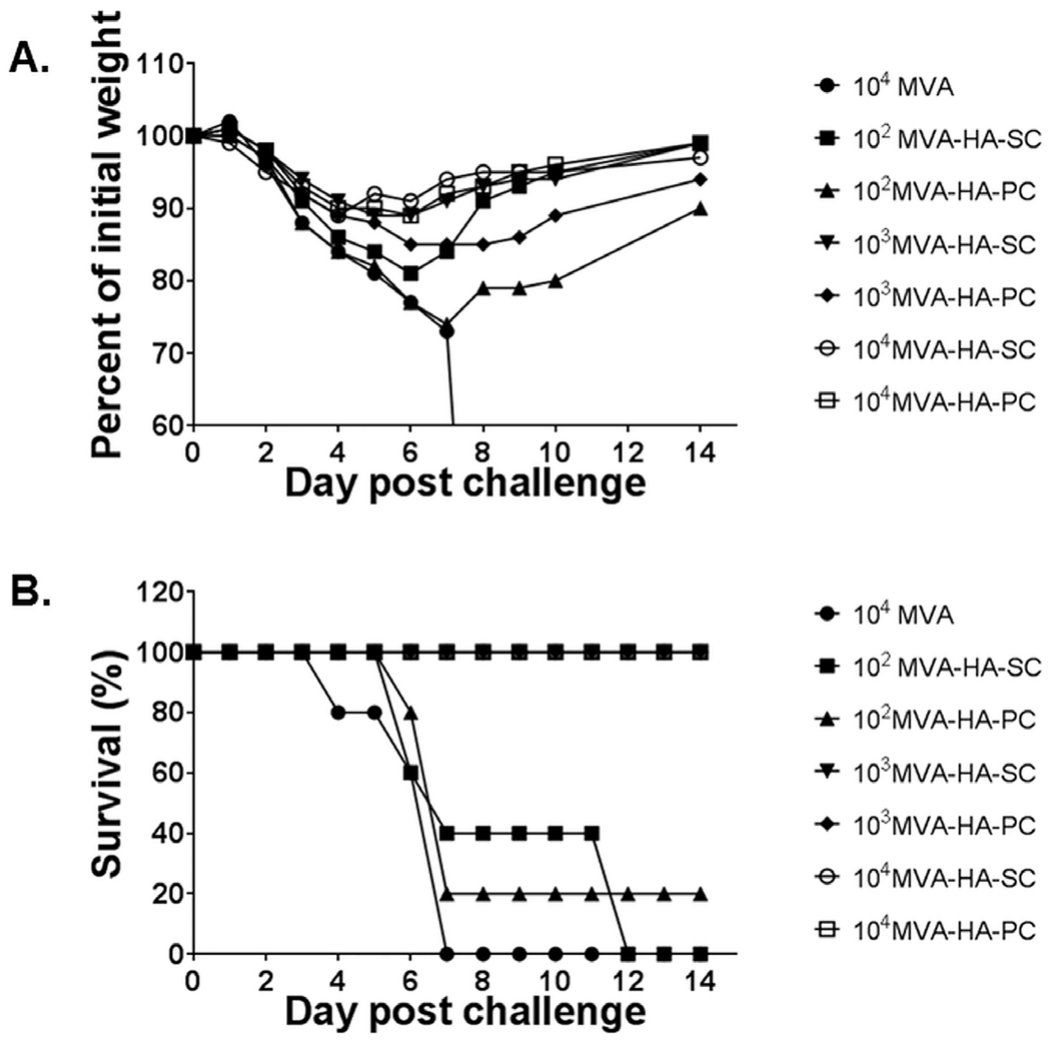
Evaluation of vaccination routes by low dose treatment with MVA-HA. Mice in groups of 5 were vaccinated with MVA or MVA-HA at the doses indicated in the legend. Booster vaccinations were administered after 3 weeks, and mice were challenged with 10^6^ pfu of influenza rgA/Viet Nam/1203/2004 (H5N1), three weeks after the second vaccination. Mice were weighed for 14 days post challenge. Weight loss as a measure of disease severity is shown in **A**, and the proportion of surviving animals in the different treatment groups are represented in **B**.

In the experiments with MVA-HA described above, all mice that were vaccinated on a prime-boost schedule with ≥10^4^ pfu of MVA-HA elicited antibody responses and protection that were indistinguishable between subcutaneous and percutaneous treatment cohorts. In order to further scrutinize the differences between the two inoculation routes, lower doses of MVA-HA were used in experiments. In one experiment, groups of mice (five per group) were vaccinated with MVA-HA at doses of 10^2^, 10^3^ or 10^4^ pfu, each dose administered via the subcutaneous or percutaneous route. A control group was inoculated subcutaneously with 10^4^ pfu of MVA. Booster inoculations in the same amount of MVA-HA or MVA were administered three weeks after mice were primed. At three weeks after the booster doses, mice were challenged with attenuated rgA/Viet Nam/1203/2004, at 10^6^ pfu per mouse, and were evaluated daily for two weeks as described above. None of the animals vaccinated with MVA survived. All mice vaccinated with ≥10^3^ pfu MVA-HA in this prime/boost schedule survived. In the 10^2^ pfu MVA-HA group, 2/5 and 1/5 survived in the subcutaneous and percutaneous groups, respectively. Influenza virus pathogenesis in these mice, as measured by weight loss (Fig 4) shows a dose-response with respect to MVA-HA, with weight loss being inversely proportional to the dose of MVA-HA. There were no major differences in survival rates between mice in the subcutaneous and percutaneous groups inoculated with the same dose of MVA-HA. At 10^2^ pfu of MVA-HA, the mean weight loss was higher in the percutaneous group than the subcutaneous group, but the difference was not statistically significant (two-tailed p-value = 0.084).

**Fig 5:**
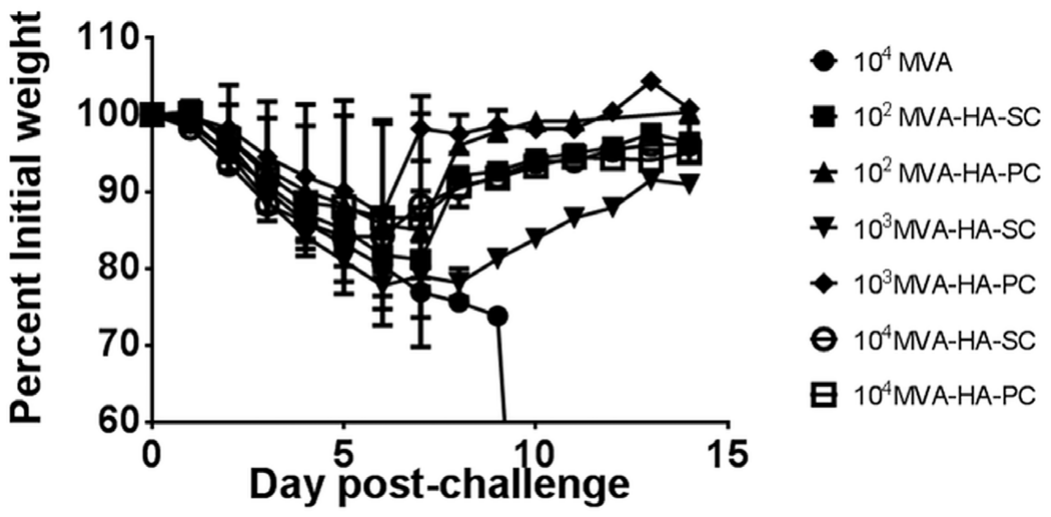
Pathogenesis of influenza rgA/Viet Nam/1203/04 virus after single low dose vaccination via the SC and PC routes. Groups of mice were vaccinated with a single dose of MVA-HA at doses of 10^2^, 10^3^, or 10^4^ pfu, via the subcutaneous and percutaneous routes. A control group was vaccinated subcutaneously with 10^4^ pfu of MVA vector. All mice were challenged with influenza rgA/Viet Nam/1203/2004 virus three weeks after vaccination and weighed daily for 2 weeks. Mean weight changes after challenge are shown. Each data point is the average of two independent experiments. Error bars represent standard deviation.

Finally, in two independent experiments, mice in groups of five in each experiment were vaccinated with low doses of MVA-HA that were administered once, and were challenged three weeks after vaccination. Groups of mice were inoculated at doses of 10^2^, 10^3^, or 10^4^ pfu of MVA-HA subcutaneously or percutaneously. Control groups received 10^4^ pfu of MVA subcutaneously. A summary of the number of surviving mice is presented in Table 4. None of the mice vaccinated with MVA survived. Among mice in the MVA-HA subcutaneous vaccination cohort, 3/10, 5/10, and 8/10 survived in the 10^2^ pfu, 10^3^ pfu, and 10^4^ pfu vaccination groups, respectively. Similarly, among mice in the percutaneous vaccination cohort, 5/10, 6/10, and 8/10 survived viral challenge. The mean weight loss among mice in these experiments are shown in Fig 5. Statistical comparisons of the number of surviving mice show that differences in survival between mice that were vaccinated with 10^4^ pfu of MVA-HA (irrespective of vaccination route) and the MVA control group, were statistically significant (Table 4). However, a comparison of the observed differences in survival between the percutaneous and subcutaneous groups at the 10^2^ pfu dose level indicates the difference is not statistically significant. This set of data suggests that even at low vaccination doses, differences between mouse groups vaccinated via the subcutaneous and percutaneous routes are not apparent in this challenge model.

**Table 4:** Protection of mice following single dose percutaneous or subcutaneous vaccination with MVA-HA.

Group	A^a^^,^^b^^,^^c^	B^d^	C	D	E	F	G
**Treatment**	10^4^ MVA	10^2^ MVA-HA-SC	10^2^ MVA-HA-PC	10^3^ MVA-HA-SC	10^3^ MVA-HA-PC	10^4^ MVA-HA-SC	10^4^ MVA-HA-PC
**Total No. of Mice**	10	10	10	10	10	10	10
**No. of Survivors**	0	3	5	5	6	8	8

^*a*^group A vs B, *p* = 0.726;

^*b*^group A vs F, *p* = 0.029;

^c^group A vs G, *p* = 0.019;

^*d*^group B vs C, *p* = 0.563

## Discussion

The modified vaccinia virus Ankara (MVA) is licensed in Europe and Canada as a third generation smallpox vaccine, and currently in clinical development for licensure in the United States. The relatively better safety record of MVA compared to first and second generation smallpox vaccines is well documented. This, in addition to its large capacity to accommodate heterologous genes, express encoded proteins, and elicit both humoral and cell-mediated immune responses also makes MVA an attractive vector for the delivery of several candidate vaccines for a variety of infectious and non-infectious human and veterinary diseases [56,57,58,59,60]. Evidence for the delivery of antigens through the skin in Asia dates back to the 1500s with the practice of variolation and continued with the advent of the smallpox vaccine in the late 18^th^ century [3]. Thus replication-competent smallpox vaccines, including those that were used in the successful eradication of smallpox, such as Dryvax, Lister, LIVP, Temple of Heaven, and EM-63, were mostly administered by skin scarification [61]. The current US-licensed second-generation smallpox vaccine, ACAM2000, is also administered by skin scarification, a procedure that is believed to be partly responsible for the success of the global eradication of smallpox by provoking robust innate and adaptive immune responses [62].

Due to the severe attenuation of MVA, as epitomized by its inability to replicate productively in many mammalian cells [11,12,13], MVA and MVA-vectored vaccines are usually administered via routes other than percutaneous in preclinical studies. Clinical investigations of MVA-vectored vaccines have mostly used intramuscular [23,34,35,36] and intradermal routes [38,39], and to a lesser extent, the subcutaneous injection route [63]. Local reactogenicity following vaccination with MVA or MVA-vectored vaccines is believed to be more severe with subcutaneous and intradermal inoculations than via intramuscular route [57,63,64]. In a comparison of the safety and immunogenicity of an MVA-vectored HIV vaccine, individuals vaccinated with MVA.HIVA by the subcutaneous and intradermal routes were found to develop more severe local reactions than those vaccinated via the intramuscular route [63]. However, intramuscular and subcutaneous tissues have relatively fewer antigen presenting cells than the skin tissue and may not be adequate for optimal immune responses [65].

Administering vaccines against infectious diseases through the skin has generated significant interest in recent years, including its use in the delivery of the BCG tuberculosis vaccine [66], and has been further boosted by the development of the microneedle patch technology, which delivers vaccines intradermally. Microneedle inoculation of vaccines has been used in preclinical evaluation of several vaccines, including inactivated polio vaccine [67], influenza vaccine [68,69,70], and measles vaccine [71]. Clinical application of vaccines to the skin has also been documented for a number of vaccines, including influenza vaccine [72,73], and rabies vaccine [74]. Recent studies have suggested that vaccine delivery through the skin takes advantage of the abundant presence of skin-resident antigen-presenting cells, including different subsets of dendritic cells and Langerhans cells, as well as infiltrating antigen presenting cells, to provoke robust immune responses that include both humoral and cell-mediated immune responses [65], and induce long-lived CD8^+^ T cell memory [75,76]. Moreover, data on the delivery of different types of vaccines through the skin suggest that both live vaccines and subunit vaccines can be administered through the skin, with successful immunization outcomes [67–71, 73,74].

Earlier data from our laboratory [17] as well as from clinical trials [57,77] suggest that delivery of the modified vaccinia virus Ankara into the intradermal layer of the skin elicited robust immune responses that were higher than intramuscular or subcutaneous inoculations, and protected mice from intranasal challenge with vaccinia virus [17]. We further showed that a severely attenuated recombinant vaccinia virus that fails to form visible plaques in several mammalian cell lines, reminiscent of MVA, elicited protective immune responses when used to vaccinate mice by scarification [46]. In the work described here, we expanded our investigation on the delivery of MVA as well as MVA-vectored antigens through the skin. In a preliminary experiment, we observed that IgG titers were higher in mice that received 10^6^ pfu of MVA by skin scarification than in mice that received the same dose of MVA by intramuscular inoculation. In subsequent experiments, antibody responses and protection of mice that were vaccinated with MVA subcutaneously or by tail scarification were higher than in those vaccinated via the intramuscular route. Melamed et al. [47] compared the antibody response elicited in response to MVA and two recombinant MVAs that had been genetically modified to replicate in Vero and BSC-1 cells, after inoculating mice by intramuscular injection or tail scarification. Their data indicate that tail scarification was efficient at inducing an antibody response, although the intramuscular route elicited higher geometric mean titers of antibody and conferred higher survival rates. The difference between their observation and the one reported here may be due to differences in experimental procedures and/or assay methods. For instance, in our work, MVA for tail scarification is typically in 2 μL volume per dose, making it easier to handle than the 10μL used by Melamed et al [47]. In subsequent experiments, the route comparison was limited to subcutaneous versus percutaneous routes, since the subcutaneous route is more commonly used in vaccination studies of MVA vectors. As the utility of MVA as a viral vector for the expression of heterologous antigens is expanding [22–40,78], we also compared the antibody responses and protection conferred by vaccination with two MVA recombinants, one expressing the HSV-2 glycoprotein D, and the other expressing the H5 hemagglutinin of influenza virus rg/A Viet Nam/1203/2004 (H5N1).

Mice that were vaccinated with MVA-gD2 by tail scarification, elicited higher than or similar titers of HSV-2 gD2-specific IgG and neutralizing antibodies to those vaccinated by subcutaneous inoculation. The observed differences in IgG titers were not statistically significant between the two routes at 10^6^ or 10^7^ pfu, but were statistically significant at 10^5^ pfu, suggesting that the percutaneous delivery of MVA-gD2 may be more effective than subcutaneous inoculation in eliciting HSV-2 neutralizing antibodies at lower vaccine doses. Consistent with previous reports [48,49], percutaneous inoculation of MVA-gD2 also elicited cell-mediated immune responses, as evident in the secretion of IFN-γ and IL-2 by re-stimulated immune splenocytes.

MVA vectors expressing influenza antigens have been shown to elicit protective immune responses in animal models [79], including mice [80–83], ferrets [84] and macaques [85,86]. In the second recombinant MVA vaccine model, MVA-HA, expressing influenza virus H5, was used to vaccinate mice by subcutaneous injection or by tail scarification, and the H5-specific antibody response and protective effectiveness against intranasal challenge with the homologous attenuated influenza virus rgA/Viet Nam/1203/2004, were assessed. The data indicate that comparable levels of antibody titers and protection were conferred by the two immunization routes. Interestingly, higher survival rates among mice vaccinated by tail scarification were recorded at low vaccine doses. Among the advantages attributed to skin delivery of vaccines is the possibility of antigen dose sparing [70,72]. The data described in this manuscript support these earlier reports as lower doses of MVA or MVA-gD2 or MVA-HA were found to elicits full or partial protection of mice.

In summary, we showed that MVA, and recombinant MVA vectors expressing HSV-2 gD2 or influenza virus H5 elicited protective immune responses in the mouse model. Taken together, the data presented in this work suggest that MVA and MVA-vectored vaccines can be effective when delivered through the skin. With the advantage that antigen delivery to the skin requires a volume that is at least 25 times (in murine models) to 200 times (in humans) less than the volume used in subcutaneous vaccination, a more comprehensive investigation of the clinical benefit of delivering MVA and MVA-vectored vaccines through the skin is necessary. Apart from its efficiency in provoking robust immune responses, it may also help to ameliorate or obliterate some of the commonly reported volume-related local reactions associated with subcutaneous or intramuscular vaccine delivery, such as pain at the site of injection, and may be more acceptable in people with needle phobia, thus enhancing compliance with scheduled immunization programs. Although the preclinical evaluation of vaccines by skin scarification, as described in our study, involves the use of improvised needles for the scarification process prior to the application of vaccines, advances in the development of the microneedle patch [87,88,89] should facilitate painless application of vaccines to the skin without the use of hypodermic injection needles or the bifurcated needle used in administering smallpox vaccines. In preclinical studies, microneedle delivery of MVA-vectored vaccines has been shown to be effective in eliciting robust immune responses against malaria [90] that are comparable to the levels attained by intradermal vaccination. Thus with further refinement, the use of microneedle delivery appears to hold a promising future for the application of viral-vectored vaccines through the skin.

## Supporting Information

S1 Fig IgG response to MVA inoculated intramuscularly or by tail scarification. Mice in groups of five were vaccinated with 10^6^ pfu of MVA via the intramuscular route (IM) or by tail scarification (PC). Serum samples obtained at day-5, day-13, day-20, and day-27 post-vaccination, were tested for vaccinia-specific IgG by ELISA (**A**). Each data point represents the mean OD_405_ value for the five mice in each treatment group. Error bar represents the standard deviation. At four weeks post-vaccination, mice were challenged with 25 LD_50_ of VV-WR, intranasally. The percentages of surviving mice are shown (**B**).
(TIF)

S2 Fig IgG response to MVA-HA and protection of vaccinated mice. Mice (5 per group) were vaccinated subcutaneously or percutaneously with 10^5^, 10^6^, or 10^7^ pfu of MVA-HA by prime-boost at an interval of 3 weeks between vaccinations. A control group received 10^7^ pfu of MVA prime-boost, subcutaneously. Serum samples obtained 3 weeks after priming (week-3) and 3 weeks after boosting (week-6) were tested for H5-specific IgG (**A**). Error bars represent standard deviation. Mice were subsequently challenged with 10^6^ pfu of influenza rgA/Viet Nam/1203/2004, and weighed daily for two weeks (**B**). A “**+**” sign represents a mouse that succumbed to infection.
(TIF)
