# Supplementary figures and images for: Percutaneous Vaccination as an Effective Method of Delivery of MVA and MVA-Vectored Vaccines
Source: PLoS One. 2016 Feb 19;11(2):e0149364. doi: 10.1371/journal.pone.0149364 (PMC4760941; doi:10.1371/journal.pone.0149364)

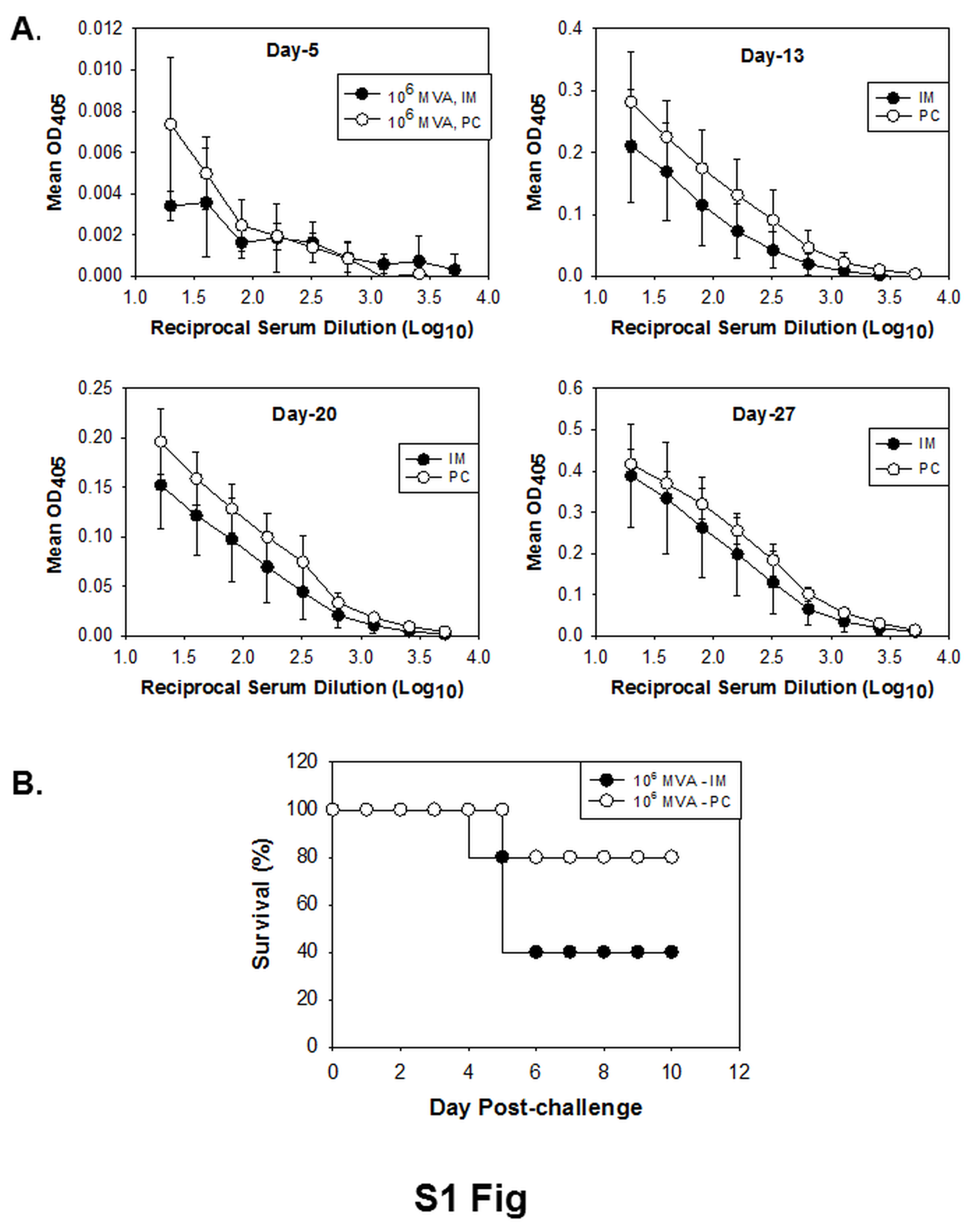

Supplement: S1 Fig — Mice in groups of five were vaccinated with 106 pfu of MVA via the intramuscular route (IM) or by tail scarification (PC). Serum samples obtained at day-5, day-13, day-20, and day-27 post-vaccination, were tested for vaccinia-specific IgG by ELISA (A). Each data point represents the mean OD405 value for the five mice in each treatment group. Error bar represents the standard deviation. At four weeks post-vaccination, mice were challenged with 25 LD50 of VV-WR, intranasally. The percentages of surviving mice are shown (B). (TIF) [file pone.0149364.s001.tif]

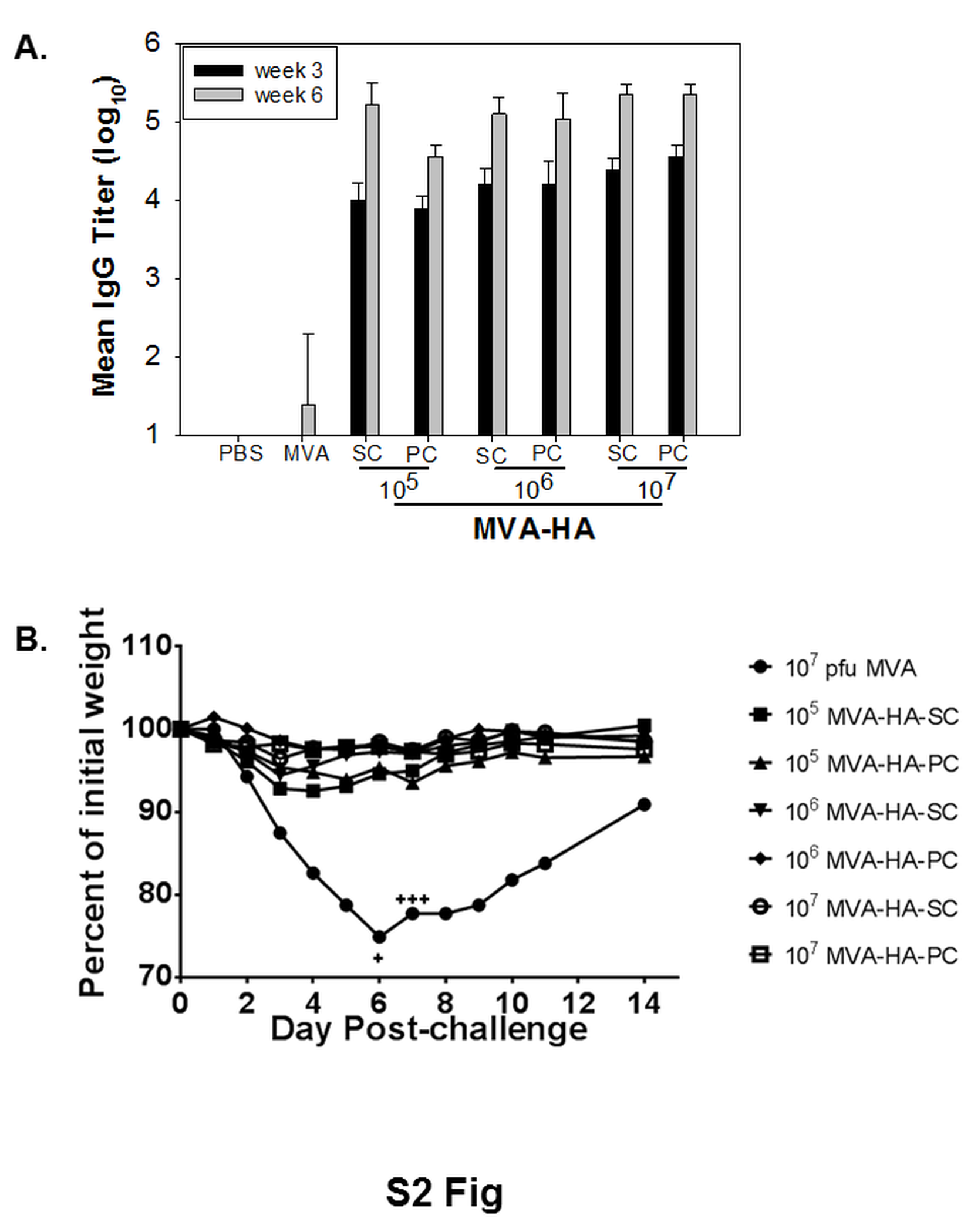

Supplement: S2 Fig — Mice (5 per group) were vaccinated subcutaneously or percutaneously with 105, 106, or 107 pfu of MVA-HA by prime-boost at an interval of 3 weeks between vaccinations. A control group received 107 pfu of MVA prime-boost, subcutaneously. Serum samples obtained 3 weeks after priming (week-3) and 3 weeks after boosting (week-6) were tested for H5-specific IgG (A). Error bars represent standard deviation. Mice were subsequently challenged with 106 pfu of influenza rgA/Viet Nam/1203/2004, and weighed daily for two weeks (B). A “+” sign represents a mouse that succumbed to infection. (TIF) [file pone.0149364.s002.tif]
